# Traditional Chinese medicine derived exosome-like nanovesicles in wound repair and skin regeneration

**DOI:** 10.3389/fcell.2025.1680757

**Published:** 2025-09-17

**Authors:** Kang Wang, Zi-Ting Yang, Fei Wang, Yun-Qi Ma, Yong Qing, Zhen-Yu Zhang

**Affiliations:** ^1^ Department of Plastic and Burn Surgery, West China Hospital, West China School of Medicine, Sichuan University, Chengdu, China; ^2^ Program of Traditional Chinese Medicine Health Preservation, Second Clinical Medical College, Henan University of Chinese Medicine, Zhengzhou, China; ^3^ Technology and Media University of Henan Kaifeng, Kaifeng, China

**Keywords:** traditional Chinese medicine, exosome-like nanovesicles, skin regeneration, wound healing, anti-inflammatory nanocarriers, angiogenesis, oxidative stress modulation

## Abstract

Skin injuries, including acute wounds, burns, and chronic ulcers, pose significant clinical challenges due to their potential to cause delayed healing and functional impairment. Exosome-like nanovesicles (ELNVs) derived from traditional Chinese medicinal (TCM) herbs have recently emerged as promising natural agents for skin repair and regeneration. These nanoscale vesicles combine the structural advantages of plant-derived delivery systems with the inherent pharmacological activities of TCM phytochemicals, offering dual roles as both bioactive agents and therapeutic carriers. Accumulating evidence indicates that TCM-derived ELNVs modulate key processes in wound healing, including inflammation resolution, fibroblast and keratinocyte activation, angiogenesis, and oxidative stress reduction. Moreover, certain vesicles have demonstrated potential in promoting hair follicle regeneration and protecting against photoaging, further highlighting their relevance in functional skin restoration. Compared with vesicles from common edible plants, TCM-ELNVs benefit from standardized cultivation, well-established traceable sourcing systems, and consistent phytochemical profiles, enhancing their suitability for therapeutic development. This review summarizes recent progress in the characterization, biological functions, and preclinical applications of TCM-derived ELNVs in cutaneous healing. Special attention is given to their mechanisms of action and their potential to serve as platforms for drug delivery and regenerative therapies. Overall, TCM-ELNVs represent a promising class of bioactive nanovesicles with broad translational potential in wound repair and skin regenerative medicine.

## 1 Introduction

**FIGURE 1:**
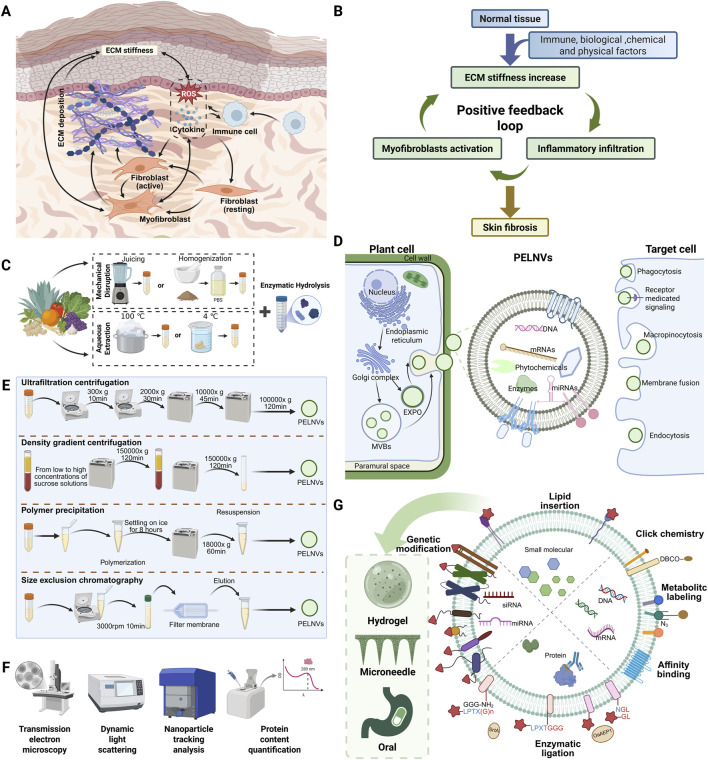
Mechanisms of skin scarring and the preparation, characterization, modification, and engineering of PELNVs. **(A)** Cellular interactions during the process of skin fibrosis. **(B)** Positive feedback between mechanical and inflammatory signals leading to ECM deposition and scar formation. **(C)** Preprocessing steps prior to PELNVs isolation. **(D)** Biogenesis, uptake, and composition of PELNVs. PELNVs are secreted by plant cells through two major pathways: the multivesicular body (MVB) pathway and the exocyst-positive organelle (EXPO) pathway. These nanovesicles carry diverse bioactive cargos, including proteins, nucleic acids (DNA, mRNA, miRNA), and plant-derived secondary metabolites. Once released, they can be internalized by recipient cells via multiple uptake mechanisms, thereby mediating a wide range of biological effects. **(E)** Commonly used isolation techniques for PELNVs, typically based on differences in physical properties such as size and density. **(F)** Representative approaches for the characterization of PELNVs. **(G)** Potential strategies for drug loading, membrane modification, and vesicle delivery using PELNVs (Liu Q. et al., 2023).

Injuries to the skin present major clinical challenges by leading to impaired healing, infection, or scarring (Peña and Martin, 2024; Jeschke et al., 2023). The process of cutaneous wound repair is orchestrated by a dynamic and tightly regulated cascade, involving oxidative stress, inflammatory responses, extracellular matrix (ECM) remodeling, fibroblast activation, and angiogenesis (Wang K. et al., 2023). Disruption of this sequence often results in pathological outcomes such as chronic non-healing wounds or excessive scar formation, both of which severely compromise patients’ quality of life. As illustrated in Figure 1A, oxidative stress and immune cell infiltration create a pro-inflammatory microenvironment that promotes fibroblast-to-myofibroblast transition and excessive ECM deposition, ultimately leading to tissue stiffness and fibrosis (Wang K. et al., 2023). Figure 1B highlights the feedback loops between pathological mechanical and immune microenvironments that perpetuate skin fibrosis and hinder regenerative outcomes (Wang K. et al., 2023).

Over the past decade, exosomes (membrane-bound extracellular vesicles typically 30–200 nm in diameter) have gained considerable attention for their regenerative capabilities (Kalluri et al., 2020). Exosomes derived from mammalian cells have demonstrated promising effects in promoting wound healing through intercellular communication and the delivery of bioactive molecules such as proteins and nucleic acids (Pegtel and Gould, 2019; Yu et al., 2024). However, the clinical application of mammalian exosomes is constrained by several limitations, including immunogenicity, potential pathogen contamination, high production costs, and variability between batches (Zheng M. et al., 2024; Chu et al., 2024). As an alternative, plant-derived exosome-like nanovesicles (PELNVs) have emerged as a natural and biocompatible vesicle system with regenerative potential. PELNVs have been shown to possess anti-inflammatory, antioxidant, and wound-healing properties (Cao et al., 2023; Wang F. et al., 2025). Compared to mammalian exosomes, plant-derived exosomes offer superior oral bioavailability, scalable production, and eliminated zoonotic transmission risks (Chu et al., 2024).

Among the diverse sources of PELNVs, exosome-like vesicles derived from traditional Chinese medicinal herbs (TCM-ELNVs) represent a particularly promising subclass. Unlike many edible plants that primarily offer nutritional benefits, TCM herbs have well-documented pharmacological activities rooted in centuries of clinical practice and modern pharmacological studies (Yang et al., 2016). Furthermore, the production of TCM-ELNVs is often more consistent and reproducible due to the long-established sourcing and quality control systems inherent to traditional Chinese medicine (Zhang R. et al., 2021; Bilia et al., 2025). Unlike cell-based systems that are sensitive to culture conditions, TCM plant materials are often derived from well-characterized strains cultivated under defined agronomic practices. As a result, the composition and bioactivity of TCM-ELNVs can be more stable across batches, which is a critical factor for their potential clinical application. Despite their therapeutic promise, the use of TCM-ELNVs in cutaneous wound healing is still in its early stages. This mini review provides an overview of the current researches on the biogenesis, composition, and functional mechanisms of TCM-ELNVs. It also summarizes recent advances in their extraction, modification, and therapeutic delivery, while highlighting the challenges and opportunities for their application in skin repair and regenerative dermatology.

## 2 Composition of TCM-ELNVs

PELNVs have been successfully isolated from a broad range of plant sources, including fruits, vegetables, roots, and seeds (Figure 1C) (Sha et al., 2024). As illustrated in Figure 1D, PELNVs are believed to originate from the multivesicular bodies within plant cells (Cui et al., 2016; Wang Y. et al., 2023). During vesicle formation, various intracellular components such as small RNAs, secondary metabolites, enzymes, and lipids are selectively loaded into the nanovesicles (Yi et al., 2023; Ly et al., 2023).

Despite the diversity in botanical origin and molecular composition, these vesicles generally share key structural characteristics, such as a lipid bilayer membrane, spherical morphology, and the capacity to encapsulate and deliver bioactive cargos (Shinge et al., 2022). It is important to note, however, that research specifically focused on TCM-ELNVs remains limited. Therefore, considering that TCM-ELNVs are essentially a subset of PELNVs, the following section summarizes the general characteristics of PELNVs, which can be intended to serve as a foundational reference for understanding and advancing research on TCM-ELNVs.

### 2.1 Lipid and membrane composition

The membrane of PELNVs is rich in plant-specific lipids such as phosphatidic acid (PA), phosphatidylcholine (PC), digalactosyldiacylglycerol (DGDG), and monogalactosyldiacylglycerol (MGDG) (Subha et al., 2023). These lipids play essential roles in vesicle stability, membrane fusion, and cellular uptake. PA in particular has been shown to enhance gastrointestinal absorption and modulate immune interactions (Subha et al., 2023), while DGDG and MGDG contribute to the fluidity and structural resilience of the lipid bilayer (Che et al., 2025). The lipid profile also supports vesicle stability under acidic and enzymatic conditions, making oral or topical application feasible (Che et al., 2025).

### 2.2 Protein and enzymatic content

Although the proteomic profile of PELNVs is less thoroughly characterized compared to their mammalian counterparts, recent mass spectrometry analyses have revealed a diverse array of protein classes within PELNVs (Che et al., 2025). These proteins can generally be categorized into transmembrane proteins and other membrane-associated proteins. The specific composition varies significantly depending on the plant species, but commonly identified proteins include aquaporins, heat shock proteins, metabolic enzymes, signaling molecules, and adhesion-related factors. These proteins are believed to play critical roles in mediating the biological and pharmacological activities of PELNVs (Dad et al., 2021).

### 2.3 Nucleic acids and small RNAs

Small RNAs, particularly microRNAs, are important functional cargos within ELNVs. These RNAs can modulate gene expression in mammalian cells, demonstrating cross-kingdom regulatory effects. Studies have identified plant-derived miRNAs involved in suppressing inflammatory pathways, modulating macrophage polarization, and regulating collagen metabolism (Wei et al., 2023; Yang et al., 2025; Li et al., 2025). The encapsulation of microRNAs within vesicles protects them from degradation, allowing functional delivery to recipient skin cells or immune cells in the wound microenvironment.

### 2.4 Phytochemical enrichment

A key distinguishing feature of TCM-ELNVs is their high content of endogenous phytochemicals. Exosome-like nanovesicles derived from Camellia sinensis flowers are rich in polyphenols and flavonoids, including epigallocatechin gallate (EGCG), epicatechin gallate (ECG), and epicatechin (EC) (Chen et al., 2022). Vesicles isolated from Zingiber officinale contain abundant 6-gingerol and 6-shogaol (Zhang et al., 2016), while Panax ginseng-derived vesicles have been shown to encapsulate bioactive ginsenosides (Cao et al., 2019). These compounds are known for their well-established pharmacological activities, such as anti-inflammatory.

## 3 Isolation, modification, and delivery strategies of TCM-ELNVs

The successful therapeutic application of TCM-ELNVs depends not only on their intrinsic biological activities but also on the ability to efficiently isolate, engineer, and deliver these vesicles to target tissues. Due to their plant origin, the extraction and formulation of TCM-ELNVs present unique advantages and challenges compared to mammalian vesicle systems. This section summarizes current approaches to isolation and purification, strategies for vesicle engineering, and methods of delivery with relevance to cutaneous applications.

### 3.1 Isolation and purification techniques

The most widely used methods for isolating PELNVs (including TCM-ELNVs) are adapted from protocols used for mammalian extracellular vesicles. These primarily include differential centrifugation, ultracentrifugation, and density gradient centrifugation. In brief, plant materials such as root juice, decoction extract, or apoplastic fluids are first clarified by low-speed centrifugation, followed by high-speed spins to concentrate vesicles as illustrated in Figures 1C,E (Huang et al., 2021; Liu et al., 2024). Final purification is often achieved using sucrose or iodixanol density gradients to remove residual debris and co-precipitated proteins (Huang et al., 2021). When isolating ELNVs from TCM herbs, several practical considerations can influence yield and purity. The presence of abundant polysaccharides, secondary metabolites, and essential oils in certain herbal materials may interfere with sedimentation efficiency or cause vesicle aggregation. To address this, enzymatic pretreatments (e.g., pectinase or cellulase) have been introduced as complementary or alternative strategies (Figure 1C) (Zhao et al., 2023). Additional processing methods can be further referenced in the review by Jung et al. (2025).

### 3.2 Characterization of vesicle properties

Once isolated, TCM-ELNVs are typically characterized by transmission electron microscopy (TEM), dynamic light scattering (DLS), and nanoparticle tracking analysis (NTA) to assess their morphology, size distribution, and concentration (Figure 1F) (Matloob et al., 2024). Additional characterization involves zeta potential measurement, protein content quantification, and RNA analysis (Matloob et al., 2024; Gao et al., 2025). Surface markers are more variable and less defined compared to mammalian exosomes; thus, functional characterization (e.g., cellular uptake, bioactivity assays) often substitutes for strict molecular marker identification (Rashidi et al., 2025; Huang Z. et al., 2024).

### 3.3 Surface modification and vesicle engineering

To enhance the therapeutic performance of TCM-ELNVs, various strategies have been developed to modify their surface properties or to encapsulate functional cargos. One common approach involves chemical conjugation of specific ligands onto the vesicle membrane to improve cellular uptake or tissue specificity (Figure 1G). For instance, vesicles may be functionalized with folic acid, peptides, or antibodies that recognize markers on keratinocytes or activated fibroblasts (Matloob et al., 2024; Liu and Su, 2019). Another strategy focuses on cargo loading, whereby therapeutic agents such as small-molecule drugs, phytochemicals, or RNA molecules are introduced into the vesicles. This can be achieved through passive incubation, membrane permeabilization techniques such as sonication or electroporation, or lipid fusion (Liu and Su, 2019). Additionally, lipid components extracted from PELNVs can be reassembled into synthetic nanovesicles using classical liposome preparation methods such as thin-film hydration and membrane extrusion (Loureiro et al., 2017; Zhuang et al., 2016; Wang et al., 2013).

### 3.4 Delivery strategies for skin repair

Effective delivery of PELNVs to injured skin tissue is critical for their therapeutic efficacy (Figure 1G). Among the available approaches, direct topical application is the most practical and noninvasive route for treating surface wounds. PELNVs can be incorporated into hydrogel matrices, thermosensitive creams, or polymer-based wound dressings to improve their adhesion, stability, and bioavailability on the skin surface (Wang Z. et al., 2024; Zheng Y. et al., 2024; Weng et al., 2025). Enhancements in skin penetration may be achieved through adjunctive technologies such as microneedle-assisted delivery (Li et al., 2024; Zheng et al., 2025). Oral administration has also been investigated in the broader context of plant-derived vesicles (Gong et al., 2024). The structural resilience of PELNVs enables them to survive gastrointestinal transit and deliver bioactive compounds to systemic circulation or distant tissues (Di et al., 2023). Oral delivery may be advantageous in conditions where skin pathology is linked to systemic inflammation or immune dysregulation (Jin et al., 2024).

## 4 Biological functions and preclinical applications of TCM-derived ELNVs in skin repair

The process of skin repair is a complex and highly coordinated biological sequence involving hemostasis, inflammation, cellular proliferation, angiogenesis, ECM remodeling, and re-epithelialization (Takeo et al., 2015; Werner and Grose, 2003). TCM-ELNVs have shown diverse therapeutic effects throughout these stages, including immunomodulation, promotion of angiogenesis, oxidative stress reduction, and stimulation of skin cell proliferation (Wu et al., 2024). Table 1 summarizes the functions and preclinical applications of TCM-ELNVs in wound repair and skin regeneration.

**TABLE 1:** Selected TCM-ELNVs and their demonstrated functions and applications in Wound Repair and Skin Regeneration**.** This table summarizes the primary biological functional mechanisms and reported therapeutic applications of some exosome-like nanovesicles derived from Traditional Chinese Medicine. These nanovesicles predominantly promote wound repair and regeneration, with some also exhibiting protection against photoaging.

Source herb (TCM)	Major functions/mechanisms	Reported applications/diseases
*Panax ginseng*	Immunomodulation, antioxidant, pro-proliferative; collagen synthesis; anti-pigmentation	Wound healing, photoaging, hyperpigmentation, hair follicle regeneration
Zingiber officinale (Ginger)	Anti-inflammatory, ROS scavenging, angiogenesis; drug delivery platform	Wound repair, antimicrobial dressing, anti-tumor adjunct
Allium sativum (Garlic)	Antimicrobial, immunoregulation (IL-17, NRF2), Wnt/β-catenin activation	Psoriasis, infected wounds, scar-free healing, hair regeneration
*Aloe vera*	Antioxidant, anti-inflammatory, pro-angiogenic; Nrf2/ARE pathway activation	Wound repair, UV-induced photoaging
*Dendrobium officinale*	Angiogenesis promotion, Akt/eNOS pathway activation, ECM remodeling	Full-thickness wound healing
*Triticum aestivum* (Wheat)	Endothelial/epithelial/fibroblast migration and proliferation	Restore epidermal homeostasis, reinforce skin barrier
*Coriandrum sativum*	Anti-inflammatory, pro-re-epithelialization, angiogenesis	Full-thickness wound healing
*Taraxacum mongolicum*	Antibacterial, collagen maturation, epithelial regeneration	Infected wounds, *S. aureus* toxin neutralization
Lycium barbarum (Goji)	Antioxidant, dermal fibroblast protection	Photoaging, UV damage repair
Vitis vinifera (Grape)	ROS modulation, dermal protection	Photoaging, skin regeneration
Solanum lycopersicum (Tomato)	Anti-inflammatory, keratinocyte/fibroblast migration	Cutaneous wound healing
Beta vulgaris (Beet)	Pro-angiogenic	Wound healing
Citrus limon (Lemon)	Macrophage polarization, fibroblast/endothelial proliferation	Healing of diabetic wounds

### 4.1 Panax ginseng

*Panax ginseng*, a cornerstone herb in traditional Chinese medicine, has been used for centuries to restore vitality, strengthen the immune system, and promote tissue recovery (Qi et al., 2011; Zhou et al., 2023). In recent years, its pharmacological effects on skin have gained increasing attention (Zhou et al., 2023; Kim et al., 2024). Experimental studies have demonstrated that ginseng enhances fibroblast proliferation, stimulates collagen synthesis, improves microcirculation, and protects skin cells from oxidative stress and ultraviolet radiation (You and Cho, 2021; Cong et al., 2023). These properties make it a promising candidate for skin regeneration therapies.

Building on this foundation, researchers have recently focused on ELNVs derived from *P. ginseng* as natural, nanoscale therapeutic systems. Ginseng-derived ELNVs have been widely studied in various disease models and are known for their strong immunomodulatory properties (Gu et al., 2024; Kim et al., 2023; Yang et al., 2024; Ma et al., 2024). In the treatment of post-inflammatory hyperpigmentation (PIH), microneedling combined with topical application of ginseng-derived exosomes significantly reduced local inflammatory cytokines and improved pigmentation in patients with acne-related PIH. In a clinical study, three sessions of microneedling followed by topical ginseng exosome application led to visibly reduced pigmentation and a more even skin tone, with no adverse effects reported (Wan et al., 2025). Additionally, ginseng-derived ELNVs have demonstrated protective effects against skin photoaging (Cho et al., 2021). These vesicles exert three primary functions (immunomodulation, anti-aging, and anti-pigmentation) by simultaneously enhancing skin immunity and improving aging phenotypes, whether intrinsic or extrinsic.

In the context of wound healing, ginseng-derived ELNVs promote cell migration and proliferation, reduce oxidative stress, suppress pro-inflammatory mediators, and stimulate collagen synthesis. Several *in vivo* and *in vitro* studies support their efficacy in accelerating skin regeneration and enhancing tissue repair (Tan M. et al., 2024; Yang et al., 2023; Choi et al., 2024; Park et al., 2025). Beyond cutaneous healing, ginseng ELNVs also support neural stem cell differentiation and peripheral nerve regeneration by modulating growth factor signaling and oxidative defense pathways (Xu et al., 2021). Moreover, ginseng-derived vesicles contribute to hair follicle regeneration, which is essential for functional skin repair. In a recent pilot intervention, daily scalp application of ginseng ELNVs over 16 weeks significantly increased the number of newly formed hairs (Aberdam et al., 2022). Taken together, ginseng-derived ELNVs not only promote wound healing but also contribute to hair follicle regeneration. These dual actions highlight their potential in functional skin regeneration. With further development, ginseng ELNVs may serve as a versatile platform for drug delivery and the restoration of skin integrity and function.

### 4.2 Zingiber officinale (ginger)

*Zingiber officinale*, commonly known as ginger, is a widely used medicinal herb in both traditional Chinese medicine and modern phytotherapy (Crichton et al., 2022). Its bioactive constituents have been reported to suppress pro-inflammatory cytokines, scavenge ROS, and promote skin wound repair (Zhang M. et al., 2021; Shaukat et al., 2023; Al-Samydai et al., 2022; Chen et al., 2012; Mokhtare and Saglam, 2025). Building upon this pharmacological foundation, ELNVs derived from ginger have emerged as a novel natural platform for disease treatment and as natural nanocarriers for drug delivery, including chemotherapy agents and RNA-based therapeutics (Pan et al., 2025; Teng et al., 2025; Huang S. et al., 2024; Guo et al., 2025; Teng et al., 2018).

In skin-related applications, ginger ELNVs have shown promise in promoting wound healing and tissue regeneration. For instance, thermosensitive hydrogels loaded with ginger-derived vesicles significantly promoted dermal fibroblast proliferation and migration and attenuated UVA-induced inflammatory damage in keratinocytes (Wang J. et al., 2024). A composite nanomembrane integrating vesicles from *Zingiber officinale*, *Aloe barbadensis*, and *Azadirachta indica*, when applied to diabetic wound models, enhanced healing outcomes and demonstrated intrinsic antibacterial effects (Miya et al., 2025). In a separate study involving osteosarcoma patients undergoing surgical excision, ginger vesicles were incorporated into a wound dressing composed of carboxymethyl chitosan methacrylate (CMCSMA) and tannic acid (TA), co-delivering doxorubicin (DOX). This therapeutic complex finally eliminate residual tumor cells, reduce reactive oxygen species (ROS), and mitigate inflammation, thereby accelerating postoperative wound closure and reducing tumor recurrence (Zhang Q. et al., 2025).

### 4.3 Allium sativum (garlic)

*Allium sativum*, commonly known as garlic, has long been valued in traditional medicine for its broad-spectrum antimicrobial, anti-inflammatory, and immunomodulatory properties (Rose et al., 2018; El-Saadony et al., 2024). It contains bioactive compounds that have demonstrated efficacy in wound healing, infection control, and tissue repair (El-Saadony et al., 2024). More recently, ELNVs derived from garlic have been investigated as a natural delivery platform capable of enhancing and concentrating these therapeutic effects. Like ginger, garlic-derived vesicles exhibit strong immunoregulatory and anti-inflammatory effects (Liu J. et al., 2023; Huang X. Z. et al., 2024; Wang X. et al., 2024). In dermatological research, garlic ELNVs have shown efficacy in treating psoriasis via dual modulation of IL-17 and NRF2 pathways, leading to reduced epidermal hyperplasia and cytokine overproduction (Kalarikkal et al., 2025).

In wound care, garlic-derived ELNVs have demonstrated intrinsic antibacterial activity against common skin pathogens. When incorporated into a hydrogel-based delivery system along with vancomycin, these vesicles effectively eradicated *Staphylococcus aureus* from infected wounds, offering a promising approach for managing antibiotic-resistant skin infections (Zhou et al., 2024). Notably, garlic vesicles have also been linked to hair follicle regeneration through activation of the Wnt/β-catenin signaling pathway and the upregulation of growth factors. These combined properties, which include antimicrobial, anti-inflammatory, and regenerative potential, suggest that garlic-derived ELNVs hold significant promise for scar-free wound healing and hair follicle restoration, warranting further investigation (Inan et al., 2023).

### 4.4 Aloe vera

*Aloe vera* is a well-known medicinal plant with a long history of therapeutic use, particularly in dermatology. Numerous studies have demonstrated that *Aloe vera* plays an important role in accelerating wound healing by promoting epithelial regeneration, modulating inflammation, and reducing oxidative stress (Liang et al., 2021). Its extracts have been formulated into various topical preparations, including gels, hydrogels, and microneedle patches, and have shown significant clinical benefits in treating burns, abrasions, and other skin injuries (Luan et al., 2024; Tehrani et al., 2022; Zhang et al., 2022; Nascimento Júnior et al., 2025).

Recent studies have identified ELNVs derived from *Aloe vera* as active components contributing to its therapeutic efficacy. These vesicles have been shown to promote the migration of human keratinocytes and dermal fibroblasts, while dose-dependently reducing intracellular reactive oxygen species (ROS) levels in keratinocytes (Kim et al., 2021). In addition, Aloe ELNVs suppress the expression of pro-inflammatory cytokines, and promote angiogenesis, suggesting their role in both early and late phases of wound healing (Kim and Park, 2022). Furthermore, in UV-induced skin aging models, topical application of Aloe ELNVs significantly reduced levels of malondialdehyde (MDA) and modulated antioxidant enzymes, including superoxide dismutase (SOD), in mouse skin tissues. These effects were mediated via activation of the Nrf2/ARE signaling pathway, highlighting their potential to protect against photoaging and oxidative damage (Sun et al., 2025). Collectively, Aloe ELNVs demonstrate multifunctional activity in skin repair, including antioxidant, anti-inflammatory, and pro-regenerative effects. Their integration into wound dressings or delivery systems may offer a promising strategy for enhancing cutaneous regeneration and delaying skin aging.

### 4.5 Other TCM-ELNVs

In addition to well-characterized herbs such as ginseng, ginger, garlic, and aloe, a number of other TCM plants have been identified as promising sources of ELNVs with potential applications in cutaneous wound healing and skin regeneration. Among them, *Dendrobium officinale*, a prized herb in Chinese pharmacopeia, is rich in polysaccharides and flavonoids that contribute to its anti-inflammatory and antioxidant effects (Zhang et al., 2023). Recent studies have shown that Dendrobium-derived nanovesicles significantly accelerate wound healing in full-thickness skin models by promoting angiogenesis, reducing IL-1β expression, activating the Akt/eNOS pathway, and enhancing ECM remodeling (Tu et al., 2024; Tu et al., 2025). Similarly, wheat-derived nanovesicles have been shown to promote the proliferation and migration of endothelial cells, epithelial cells, and dermal fibroblasts, indicating their potential in restoring epidermal homeostasis and reinforcing skin barrier function (Şahin et al., 2019).

Other edible or medicinal plants used in TCM have also demonstrated skin-healing potential. For example, ELNVs from *Coriandrum sativum* (coriander) enhanced re-epithelialization and neovascularization in whole-layer skin wound model of mice, with concurrent downregulation of inflammatory cytokines (Wang T. et al., 2025). A gel formulation incorporating *Taraxacum mongolicum* (dandelion)-derived nanovesicles was shown to neutralize *Staphylococcus aureus* exotoxins, accelerate epithelial regeneration, promote collagen maturation, and alleviate inflammation (Tan S. et al., 2024). Both grape-derived and *Lycium barbarum* (goji berry)-derived ELNVs have demonstrated potential in mitigating photoaging and promoting the repair of UV-induced skin damage (Zhang Y. et al., 2025; Wang et al., 2022; M et al., 2025). These effects are attributed particularly to their antioxidant and protective effects on dermal fibroblasts. In addition, tomato-derived ELNVs exhibit anti-inflammatory activity and accelerate wound healing by promoting the migration of keratinocytes and fibroblasts (Mammadova et al., 2023; Daniello et al., 2024). *Beta vulgaris* (beet) vesicles demonstrated pro-angiogenic effects (Mahdipour, 2022). Lemon-derived vesicles modulate macrophage polarization reprogramming and promote the proliferation and migration of endothelial cells and fibroblasts, thereby facilitating the healing of diabetic wounds (Jin et al., 2025).

## 5 Conclusion and perspective

ELNVs derived from TCM herbs hold great therapeutic potential for wound repair and skin regeneration. The nano vesicles constructed by combining the structural advantages of plant-based vesicles with the inherent biological activity of traditional Chinese medicine phytochemicals can not only serve as delivery carriers for wound treatment, but also as functional agents to promote skin regeneration. Current studies suggest that TCM-ELNVs can promote skin regeneration through anti-inflammatory, pro-proliferative, angiogenic, and antioxidant mechanisms. Compared with vesicles from common edible plants, TCM-ELNVs benefit from advantages such as standardized cultivation of source plants, well-established traceable sourcing systems, and reproducible pharmacological properties, all of which significantly enhance their translational potential. Looking ahead, several challenges and opportunities remain. First, deeper mechanistic studies are needed to link specific phytochemicals within ELNVs to their observed bioactivities, which will facilitate rational design of vesicle-based therapeutics. Second, scaling up production under good manufacturing practice (GMP) conditions and evaluating long-term safety in preclinical and clinical settings are essential for translational success. Finally, the incorporation of advanced biomaterials such as hydrogels enables the modification, loading, and targeted delivery of TCM-ELNVs, thereby enhancing their stability, bioavailability, and therapeutic efficacy in wound repair and skin regeneration. Collectively, ongoing pharmacological and technological advances are poised to transform TCM-ELNVs into promising candidates for targeted drug delivery and functional skin regeneration systems.
